# Moving towards a population health approach to the primary prevention of common mental disorders

**DOI:** 10.1186/1741-7015-10-149

**Published:** 2012-11-27

**Authors:** Felice N Jacka, Arnstein Mykletun, Michael Berk

**Affiliations:** 1 Deakin University, School of Medicine and Barwon Health, PO Box 281, Geelong, 3220, Australia; 2 The University of Melbourne, Department of Psychiatry, Parkville, 3010, Australia; 3 Norwegian Institute of Public Health, Division of Mental Health, Kalfarveien 31, 5018, Bergen, Norway; 4 University of New South Wales, School of Psychiatry, Black Dog Institute, Building Hospital Road, Prince of Wales Hospital, Randwick, 2031, Australia; 5 The Florey Institute of Neuroscience and Mental Health, Genetics Lane, Royal Parade, The University of Melbourne, Parkville, 3010, Australia; 6 Orygen Youth Health Research Centre, 35 Poplar Road, Parkville, 3052, Australia

**Keywords:** Anxiety, etiology, common mental disorders, diet, depression, lifestyle, physical activity, prevention, risk, smoking

## Abstract

There is a need for the development of effective universal preventive approaches to the common mental disorders, depression and anxiety, at a population level. Poor diet, physical inactivity and smoking have long been recognized as key contributors to the high prevalence noncommunicable diseases. However, there are now an increasing number of studies suggesting that the same modifiable lifestyle behaviors are also risk factors for common mental disorders. In this paper we point to the emerging data regarding lifestyle risk factors for common mental disorders, with a particular focus on and critique of the newest evidence regarding diet quality. On the basis of this most recent evidence, we consequently argue for the inclusion of depression and anxiety in the ranks of the high prevalence noncommunicable diseases influenced by habitual lifestyle practices. We believe that it is both feasible and timely to begin to develop effective, sustainable, population-level prevention initiatives for the common mental illnesses that build on the established and developing approaches to the noncommunicable somatic diseases.

## Background

The common mental disorders, depression and anxiety, are major public health problems across the globe. Unipolar depression now accounts for the largest burden of disability in the developed world, reducing functional capacity [1] and increasing the risk for early mortality across causes of death [2]. In many medical disorders, from infections to cancer and cardiovascular disorders, the greatest contribution to health has been through prevention rather than treatment. However, prevention at a public health level in psychiatric disorders has received less attention than individualized treatment efforts. Thus, there is a need for the development of effective universal preventive approaches to the common mental disorders at a population level [3].

One of the great complexities of the preventative paradigm in psychiatry is the sheer plethora of interacting factors of variable plasticity and reversibility. While other potentially modifiable factors, including social inequality, social networks, child abuse, and drug and alcohol abuse, play a role in depression risk, preventative efforts need to focus on areas of greatest plasticity and reach, whilst acknowledging the role of the other factors. One such plastic factor is lifestyle, a term which encompasses diet, physical activity and smoking.

Poor diet, physical inactivity and smoking have long been recognized as key contributors to the high prevalence noncommunicable diseases, such as heart disease, type 2 diabetes and cancer. However, there are now an increasing number of studies suggesting that the same modifiable lifestyle behaviors are also risk factors for common mental disorders. Growing evidence indicates a role for physical inactivity as a risk factor for common mental disorders (for example [4-6]), while exercise has been shown to be effective in treatment studies [7]. There is some evidence for smoking also independently increasing the risk for common mental disorders [8-10]. Diet quality is the most recent area of attention in the lifestyle-mental health research field. Here we make the important distinction between habitual dietary intakes and the limited and inconsistent literature on nutritional supplementation as a treatment for depression, wherein, apart from omega 3 fatty acids in severe depression [11] and folate as an adjunctive treatment [12], there is little quality evidence for effectiveness [13]. Neither will we address the literature on the dietary intake of individual nutrients and depression (for review see [14]) as these are also equivocal and likely confounded by overall dietary quality.

In this paper we will present a brief overview of the recent observational data regarding diet quality as a risk factor for common mental disorders. This new evidence base adds to what is already known about the importance of physical activity to depression and the developing evidence regarding smoking as an independent risk factor for depression; as such it can be considered 'the final piece in the lifestyle puzzle'. On the basis of this newest evidence, we will thus argue for the inclusion of depression and anxiety in the ranks of the high prevalence noncommunicable disorders influenced by habitual lifestyle practices. We will also argue for the imperative to develop universal primary prevention strategies for common mental disorders, applying the lessons of successes and failures of population health approaches that have been used for other noncommunicable disorders.

## Discussion

### What is the evidence for an association between diet quality and the common mental disorders?

In 2010, in a population-based study of over 1,000 adult women, Jacka *et al. *[15] reported that women who had a diet characterized by a higher intake of unhealthy western-type foods were more likely to have either major depression or dysthymia, while those whose diets were higher in recognized healthy foods, such as vegetables, lean meats, fish and wholegrains, were less likely to have clinical depressive or anxiety disorders. Similar associations were observed between diet quality and bipolar disorder in the same cohort [16]. Subsequent investigations in a study of more than 5,000 population-based Norwegians demonstrated that adults with better quality diets were less likely to be depressed, while a higher intake of processed and unhealthy foods was associated with increased anxiety [17]. These studies are complemented by prospective studies from other groups: data from the SUN Cohort study in Spain, based on more than 10,000 middle-aged university graduates, demonstrated a prospective inverse association over approximately four and a half years between the level of adherence to a Mediterranean dietary pattern and the risk for incident depression, and a positive association between the consumption of fast foods and commercial baked goods and depression risk [18,19]. In the Whitehall II cohort study of nearly 3,500 public servants, the authors observed an increased risk for self-reported depression for those on a diet higher in western-style foods, and reduced risk for those on a diet higher in whole foods over approximately five years of follow-up [20]. In 529 women participating in the RHEA mother and baby cohort study, women with better quality diets during pregnancy were at reduced risk for postpartum depression [21]. Numerous recent cross-sectional studies from research groups in Japan [22,23], China [24], the US [25-28] and elsewhere have supported these findings in adults. It is worth noting that these studies regarding dietary intake are also supported by studies showing that serum folate concentrations, which are markers of dietary quality, predict depression risk over time [29].

Given that adolescence is the age of risk for the onset of these disorders, it is noteworthy that similar data exist in population-based child and adolescent studies: in a 2009 study of more than 1,300 adolescents, Oddy *et al. *[30] reported that those eating a diet characterized by higher intakes of takeaway foods, red meat and confectionary had increased internalizing and externalizing behaviors, whilst the intake of fruits and vegetables showed the opposite association. In a Chinese study of more than 5,000 young adolescents, healthy dietary habits were associated with reduced psychological symptoms, whilst unhealthy dietary habits had a positive relationship with such symptoms [31]. Similarly, a study of 7,000 young population-based adolescents reported inverse relationships between measures of diet quality and the likelihood of adolescent depression, with higher scores on an unhealthy dietary measure associated with increased symptomatic depression, and higher scores on a healthy dietary measure with decreased symptomatic depression [32]. A subsequent prospective study reported that diet quality was associated with adolescent mental health over a two-year period, even after taking mental health at baseline into account [33]. Moreover, improvements in diet quality were mirrored by improvements in mental health, while reductions in diet quality were associated with declining psychological functioning over the follow-up period. More recently still, a cross-sectional study of German children participating in both the GINIplus and LISAplus cohort studies reported that higher diet quality was associated with fewer emotional symptoms, while increased confectionary consumption had a positive relationship with emotional symptomatology [34].

### How strong is the evidence for a causal effect of diet on common mental disorders?

Although the existing observational data are consistent, it is still an unresolved question as to whether the observed associations are a result of causal relationships between diet and mental health, or whether the associations between diet and common mental disorders are due to residual confounding by socioeconomic status or healthy lifestyle behaviors in general, or confounding by unrecognized third factors. Further, reverse causality is not entirely ruled out. The same methodological challenges apply to studies of an eventual effect of physical activity and smoking on mental health. The picture is further complicated by the recognition of the bidirectional relationship between diet and mental health, wherein appetite changes may be a result of mental health symptoms rather than a causative factor. Although the large majority of the previous observational studies have addressed confounding, and the prospective studies have assessed the reverse causality hypothesis, there are obvious limitations to observational studies as regards determining causality. Moreover, apart from one report of no relationship between measures of healthy diet and depression in male, but not female, college students [35] and another study of 865 women reporting no relationship between dietary patterns and postpartum depression [36], there is a dearth of negative studies. There are no published papers examining the issue of publication bias in the diet-common mental disorders literature, thus we cannot exclude publication bias in favor of positive findings.

On the other hand, it appears that the published studies on the diet-mental health association do satisfy most of the Bradford Hill criteria for causality [37]: that of consistency, with concordant findings and effect sizes across cultures, genders and age groups, with multiple methods used to assess diet quality and mental health; biological gradient [15,18,19,21,24,32,33]; temporality [18-21,33]; biological plausibility [38,39]; and coherence of the findings with what we already know about the impact of habitual diet on noncommunicable illnesses. However, we only have very preliminary experimental evidence at this time [40].

In contrast to physical activity-depression research, randomized controlled trial evidence for the diet-common mental disorders association is lacking. However, such trials, when conducted, will present myriad methodological problems, as often seen in the field of depression research, such as difficulties with blinding and the problems inherent in conducting randomized controlled trials in patients with depression [41]. Beyond such trials in clinical samples, proving the effectiveness of universal prevention is also problematic, given the substantial statistical power required [42]. However, the reduction in smoking in the general population in many western societies is clearly the result of primary prevention at a population level [43], despite the lack of evidence from randomized controlled trials. Consequently, we would argue that primary prevention holds considerable promise for reducing the burden of depression and anxiety in the population, in concert with selected and indicated prevention, as well as treatment.

### Mechanisms

Diet, exercise and smoking may influence mental health through a number of different pathways, including via modification of neurotrophins critical to depression [44,45]. Diet, smoking and exercise also influence systemic inflammation [46,47], which is thought to play a role in the genesis of depression [48]. Inflammation is a common pathophysiological mechanism thought to, at least in part, underpin the bidirectional relationships between depression and noncommunicable illnesses such as obesity [49], cardiovascular disease and type 2 diabetes [50]. This suggests immune dysregulation as a key shared pathophysiological pathway by which poor lifestyle practices contribute to both the common mental disorders and the noncommunicable disorders [51].

### Prevention: taking a population health approach to the common mental disorders

The 2010 World Health Organization Global Status Report highlights the impact of the changing environmental determinants of the noncommunicable disorders, including poor dietary and physical activity behaviors that have been driven by changes to the global food supply systems, policy, and marketing, as well as increasing urbanization [52]. It outlines and promotes population-wide preventive interventions targeting lifestyle behaviors, primarily aimed at reducing cardiovascular diseases, cancers, diabetes and chronic lung diseases. Although mental health disorders are mentioned as part of the 'broader scope of the noncommunicable conditions,' the prevention of mental health problems using these targeted lifestyle strategies is not specifically addressed. However, we believe that it is both feasible and timely to use our emerging understanding of the impact on mental health of lifestyle behaviors to begin to develop effective, sustainable, population-level prevention initiatives for the common mental disorders. The challenge is how to expand from the nascent epidemiological evidence to develop and evaluate a prevention system for lifestyle-related mental ill health.

### A strategy for moving forward

There is agreement that decreasing lifestyle-related risk must address a complex and interconnected mix of etiological factors, from individual behaviors to cultural norms and policy environments [53]. To begin to develop universal prevention approaches for the common mental disorders, it is necessary to link policy options with epidemiology and biological sciences. As such, we would argue that there is a continuing need to generate quality epidemiological evidence to address the identified shortcomings in the literature outlined above. Further research that attempts to disentangle the multitudinous interacting social, medical, behavioral, cultural and biological factors that impact upon both diet quality and mental illness risk is required, while systematic reviews of the diet-mental health observational research, with best evidence syntheses, would also be a valuable and timely contribution to this developing field.

Epidemiological studies should also gather biomarker data that elucidate the role of biological factors, such as oxidative stress, inflammation and neurotrophic mechanisms, as mediators in the relationship between lifestyle factors and the high prevalence mental illnesses. While there are data to show that diet quality, physical activity and mental health are each independently associated with neurotrophin levels and inflammatory and oxidative processes, to date there have been few studies directly addressing mediation via biological mechanisms. Such studies are necessary to either support or refute the case for causal biological relationships and to attempt to explicate the fundamental processes by which lifestyle may influence common mental disorders. There is also a pressing need for evidence from methodologically rigorous randomized controlled trials of dietary improvement as a treatment intervention in those with existing depressive illness. Although not addressing the question of prevention, such evidence - if positive - would be further support for direct causal relationships.

The next strategy advocated is to leverage on existing intervention studies in the field of obesity prevention research to assess the impact of lifestyle improvement on mental health outcomes, particularly in young people. The majority of mental health problems have their onset in childhood and adolescence [54] and this is where the potential for prevention is likely to be greatest. The recent Cochrane Review of effective strategies to prevent obesity in children found strong evidence to support beneficial effects of child obesity prevention programs on weight gain, particularly for programs targeted to children aged six to twelve years [55]. Given that the strategies employed to reduce the prevalence of obesity in children and adolescents overlap substantially with the objective of reducing the prevalence of mental health problems in children and adolescents through lifestyle improvement, the potential to leverage on existing and planned studies in this field and apply the findings to mental health prevention is clear. In this way, mental health research can avoid the failures of previous approaches to obesity prevention and build on the successes. In parallel, economic evaluations of such interventions are necessary to afford comparisons of various prevention models and the projection of the impact and cost-effectiveness of different policy and environmental changes at a population level.

Previous analyses of the costs and benefits of various approaches to public health, including the Assessing Cost-Effectiveness Prevention study [56], have identified that policy approaches to prevention are more cost-effective than health promotion or clinical interventions. As public health policy is the key responsibility of government, government must be at the forefront of prevention initiatives. Suggested priority actions overlap with those for noncommunicable diseases and include policies to improve the food and built environments and greater funding for prevention programs, keeping always in mind the impact on social equity [53].

It is also necessary to incorporate these proposed prevention strategies with attempts to address other risk factors. Childhood abuse and neglect are critical risks, however they are arguably better addressed via the justice and social welfare systems, where active programs are in play. Similarly, the issue of how society should address the regulatory and therapeutic framework of drug and alcohol abuse is of core salience to risk and prevention. Western societies are increasingly nuclear, fragmented, mobile and anomic. The lack of social connectedness is a risk for depression, and similarly merits attention. Thus, policy efforts aimed at prevention, as well as public health messages and educational programs developed in partnership with public health and advocacy groups, will need to integrate these new data regarding lifestyle with those previously understood. From a clinical perspective, support for lifestyle improvement should be routinely provided to all patients with depression and incorporated into treatment guidelines [57].

### What sort of impact can we expect to have?

It is important to acknowledge the likely limitations of lifestyle modification in the prevention of the common mental disorders in individuals. In the observational studies, diet and physical activity do not account for a large proportion of the variance in psychological symptomatology. However, there are no identified factors, other than genetics, that account for any more than a small percentage of the variance in depression. Given the size of the burden of illness, the benefits of even a minimal impact on the prevalence of depression will be substantial, as even modest benefits at the individual level accrue to the entire population. Finally, it should be acknowledged that the evidence base for lifestyle as a true causal risk factor for common mental disorders is still developing. However, the benefits to physical health of improvements in lifestyle behaviors are well-established and the precautionary principle provides justification for beginning the development of the outlined programs in the absence of unequivocal evidence.

## Summary

The identification of 'modifiable social and biological risk factors across the life course' and the need to 'advance prevention and implementation of early interventions' were recently nominated as the first two grand challenges in global mental health [58]. We hereby present the case for inclusion of the common mental disorders under the noncommunicable diseases umbrella, based on the recent identification of lifestyle as a modifiable risk factor, and call for the development of a coordinated strategy for the universal primary prevention of the common mental disorders that builds on the established and developing approaches to the noncommunicable somatic diseases.

## Competing interests

The authors declare that they have no competing interests.

## Authors' contributions

FNJ led the development and authorship of this article. AM and MB contributed equally to intellectual content and manuscript preparation. All authors have read and approved the article for publication.

## Authors' information

FNJ is a psychiatric epidemiologist and Principal Research Fellow within the School of Medicine at Deakin University, Geelong, Australia. She is also an Honorary Research fellow in the Department of Psychiatry at the University of Melbourne, Australia. AM is a psychiatric epidemiologist and Professor at the Norwegian Institute of Public Health, with academic affiliations with the University of Bergen, Norway and the University of New South Wales, School of Psychiatry, Sydney, Australia. MB is Alfred Deakin Professor of Psychiatry and the Director of the Strategic Research Centre for Psychiatry and Epidemiology within the School of Medicine, Deakin University; Professorial Research Fellow at The Florey Institute of Neuroscience and Mental Health and the Department of Psychiatry at the University of Melbourne; and leads the first episode bipolar program at Orygen Youth Health, Melbourne, Australia.

## Pre-publication history

The pre-publication history for this paper can be accessed here:

http://www.biomedcentral.com/1741-7015/10/149/prepub

## References

[B1] Mykletun A Overland S Dahl AA Krokstad S Bjerkeset O Glozier N Aaro LE Prince M A population-based cohort study of the effect of common mental disorders on disability pension awards Am J Psychiatry 2006 163 1412 1418 10.1176/appi.ajp.163.8.1412 16877655

[B2] Mykletun A Bjerkeset O Overland S Prince M Dewey M Stewart R Levels of anxiety and depression as predictors of mortality: the HUNT study Br J Psychiatry 2009 195 118 125 10.1192/bjp.bp.108.054866 19648541

[B3] Cuijpers P Beekman AT Reynolds CF Preventing depression: a global priority JAMA 2012 307 1033 1034 10.1001/jama.2012.271 22416097 PMC3397158

[B4] Lucas M Mekary R Pan A Mirzaei F O'Reilly EJ Willett WC Koenen K Okereke OI Ascherio A Relation between clinical depression risk and physical activity and time spent watching television in older women: a 10-year prospective follow-up study Am J Epidemiol 2011 174 1017 1027 10.1093/aje/kwr218 21984659 PMC3243936

[B5] Harvey SB Hotopf M Overland S Mykletun A Physical activity and common mental disorders Br J Psychiatry 2010 197 357 364 10.1192/bjp.bp.109.075176 21037212

[B6] Pasco JA Williams LJ Jacka FN Henry MJ Coulson CE Brennan SL Leslie E Nicholson GC Kotowicz MA Berk M Habitual physical activity and the risk for depressive and anxiety disorders among older men and women Int Psychogeriatr 2011 23 292 298 10.1017/S1041610210001833 20863424

[B7] Stathopoulous G Powers MB Berry AC Smits JAJ Otto M Exercise interventions for mental health: a quantitative and qualitative review Clin Psychol Sci Prac 2006 13 179 193 10.1111/j.1468-2850.2006.00021.x

[B8] Mykletun A Overland S Aaro LE Liabo HM Stewart R Smoking in relation to anxiety and depression: evidence from a large population survey: the HUNT study Eur Psychiatry 2008 23 77 84 10.1016/j.eurpsy.2007.10.005 18082377

[B9] Pasco JA Williams LJ Jacka FN Ng F Henry MJ Nicholson GC Kotowicz MA Berk M Tobacco smoking as a risk factor for major depressive disorder: population-based study Br J Psychiatry 2008 193 322 326 10.1192/bjp.bp.107.046706 18827296

[B10] Breslau N Novak SP Kessler RC Daily smoking and the subsequent onset of psychiatric disorders Psychol Med 2004 34 323 333 10.1017/S0033291703008869 14982138

[B11] Appleton KM Rogers PJ Ness AR Updated systematic review and meta-analysis of the effects of n-3 long-chain polyunsaturated fatty acids on depressed mood Am J Clin Nutr 2010 91 757 770 10.3945/ajcn.2009.28313 20130098

[B12] Taylor MJ Carney SM Goodwin GM Geddes JR Folate for depressive disorders: systematic review and meta-analysis of randomized controlled trials J Psychopharmacol 2004 18 251 256 10.1177/0269881104042630 15260915

[B13] Jorm AF Christensen H Griffiths KM Rodgers B Effectiveness of complementary and self-help treatments for depression Med J Aust 2002 176 Suppl S84 96 12065003 10.5694/j.1326-5377.2002.tb04508.x

[B14] Murakami K Sasaki S Dietary intake and depressive symptoms: a systematic review of observational studies Mol Nutr Food Res 2010 54 471 488 10.1002/mnfr.200900157 19998381

[B15] Jacka FN Pasco JA Mykletun A Williams LJ Hodge AM O'Reilly SL Nicholson GC Kotowicz MA Berk M Association between western and traditional diets and depression and anxiety in women Am J Psychiatry 2010 167 305 311 10.1176/appi.ajp.2009.09060881 20048020

[B16] Jacka FN Pasco JA Mykletun A Williams LJ Nicholson GC Kotowicz MA Berk M Diet quality in bipolar disorder in a population-based sample of women J Affect Disord 2011 129 332 337 10.1016/j.jad.2010.09.004 20888648

[B17] Jacka FN Mykletun A Berk M Bjelland I Tell GS The association between habitual diet quality and the common mental disorders in community-dwelling adults: the Hordaland Health study Psychosom Med 2011 73 483 490 10.1097/PSY.0b013e318222831a 21715296

[B18] Sanchez-Villegas A Delgado-Rodriguez M Alonso A Schlatter J Lahortiga F Majem LS Martinez-Gonzalez MA Association of the Mediterranean dietary pattern with the incidence of depression: the Seguimiento Universidad de Navarra/University of Navarra follow-up (SUN) cohort Arch Gen Psychiatry 2009 66 1090 1098 10.1001/archgenpsychiatry.2009.129 19805699

[B19] Sanchez-Villegas A Toledo E de Irala J Ruiz-Canela M Pla-Vidal J Martinez-Gonzalez MA Fast-food and commercial baked goods consumption and the risk of depression Public Health Nutr 2012 15 424 432 10.1017/S1368980011001856 21835082

[B20] Akbaraly TN Brunner EJ Ferrie JE Marmot MG Kivimaki M Singh-Manoux A Dietary pattern and depressive symptoms in middle age Br J Psychiatry 2009 195 408 413 10.1192/bjp.bp.108.058925 19880930 PMC2801825

[B21] Chatzi L Melaki V Sarri K Apostolaki I Roumeliotaki T Georgiou V Vassilaki M Koutis A Bitsios P Kogevinas M Dietary patterns during pregnancy and the risk of postpartum depression: the mother-child 'Rhea' cohort in Crete, Greece Public Health Nutr 2011 14 1663 1670 10.1017/S1368980010003629 21477412

[B22] Nanri A Kimura Y Matsushita Y Ohta M Sato M Mishima N Sasaki S Mizoue T Dietary patterns and depressive symptoms among Japanese men and women Eur J Clin Nutr 2010 64 832 839 10.1038/ejcn.2010.86 20485303

[B23] Aihara Y Minai J Aoyama A Shimanouchi S Depressive symptoms and past lifestyle among Japanese elderly people Community Ment Health J 2011 47 186 193 10.1007/s10597-010-9317-1 20455023

[B24] Liu C Xie B Chou CP Koprowski C Zhou D Palmer P Sun P Guo Q Duan L Sun X Anderson Johnson C Perceived stress, depression and food consumption frequency in the college students of China Seven Cities Physiol Behav 2007 92 748 754 10.1016/j.physbeh.2007.05.068 17585967

[B25] Kuczmarski MF Cremer Sees A Hotchkiss L Cotugna N Evans MK Zonderman AB Higher Healthy Eating Index-2005 scores associated with reduced symptoms of depression in an urban population: findings from the Healthy Aging in Neighborhoods of Diversity Across the Life Span (HANDLS) study J Am Diet Assoc 2010 110 383 389 10.1016/j.jada.2009.11.025 20184988 PMC2850196

[B26] Beydoun MA Fanelli Kuczmarski MT Beydoun HA Shroff MR Mason MA Evans MK Zonderman AB The sex-specific role of plasma folate in mediating the association of dietary quality with depressive symptoms J Nutr 2010 140 338 347 10.3945/jn.109.113878 20032481 PMC2806887

[B27] Crawford GB Khedkar A Flaws JA Sorkin JD Gallicchio L Depressive symptoms and self-reported fast-food intake in midlife women Prev Med 2011 52 254 257 21276813 10.1016/j.ypmed.2011.01.006 PMC3062726

[B28] Pagoto SL Ma Y Bodenlos JS Olendzki B Rosal MC Tellez T Merriam P Ockene IS Association of depressive symptoms and lifestyle behaviors among Latinos at risk of type 2 diabetes J Am Diet Assoc 2009 109 1246 1250 10.1016/j.jada.2009.04.010 19559144 PMC2733911

[B29] Nanri A Hayabuchi H Ohta M Sato M Mishima N Mizoue T Serum folate and depressive symptoms among Japanese men and women: a cross-sectional and prospective study Psychiatry Res 2012 in press 10.1016/j.psychres.2012.04.040 22682152

[B30] Oddy WH Robinson M Ambrosini GL O'Sullivan TA de Klerk NH Beilin LJ Silburn SR Zubrick SR Stanley FJ The association between dietary patterns and mental health in early adolescence Prev Med 2009 49 39 44 10.1016/j.ypmed.2009.05.009 19467256

[B31] Weng TT Hao JH Qian QW Cao H Fu JL Sun Y Huang L Tao FB Is there any relationship between dietary patterns and depression and anxiety in Chinese adolescents? Public Health Nutr 2012 15 673 382 10.1017/S1368980011003077 22115495

[B32] Jacka FN Kremer PJ Leslie E Berk M Patton G Toumbourou JW Williams JW Associations between diet quality and depressed mood in adolescents: results from the Healthy Neighbourhoods study Aust N Z J Psychiatry 2010 44 435 442 10.3109/00048670903571598 20397785

[B33] Jacka F Kremer PJ Berk M de Silva-Sanigorski A Moodie M Leslie E Pasco J Swinburn B A prospective study of diet quality and mental health in adolescents PLoS One 2011 6 e24805 10.1371/journal.pone.0024805 21957462 PMC3177848

[B34] Kohlboeck G Sausenthaler S Standl M Koletzko S Bauer CP von Berg A Berdel D Kramer U Schaaf B Lehmann I Herbarth O Heinrich J Food intake, diet quality and behavioral problems in children: results from the GINI-plus/LISA-plus Studies Ann Nutr Metab 2012 60 247 256 22677949 10.1159/000337552

[B35] Mikolajczyk RT El Ansari W Maxwell AE Food consumption frequency and perceived stress and depressive symptoms among students in three European countries Nutr J 2009 8 31 10.1186/1475-2891-8-31 19604384 PMC2716364

[B36] Okubo H Miyake Y Sasaki S Tanaka K Murakami K Hirota Y Dietary patterns during pregnancy and the risk of postpartum depression in Japan: the Osaka Maternal and Child Health Study Br J Nutr 2011 105 1251 1257 10.1017/S0007114510004782 21144112

[B37] Bradford Hill A The environment and disease: association or causation? Proc R Soc Med 1965 58 5 295 300 14283879 10.1177/003591576505800503 PMC1898525

[B38] Jacka FN Berk M Food for thought Acta Neuropsychiatrica 2007 19 321 323 10.1111/j.1601-5215.2007.00246.x

[B39] Milaneschi Y Bandinelli S Penninx BW Vogelzangs N Corsi AM Lauretani F Kisialiou A Vazzana R Terracciano A Guralnik JM Ferrucci L Depressive symptoms and inflammation increase in a prospective study of older adults: a protective effect of a healthy (Mediterranean-style) diet Mol Psychiatry 2011 16 589 590 10.1038/mp.2010.113 21042319 PMC5003408

[B40] Imayama I Alfano CM Kong A Foster-Schubert KE Bain CE Xiao L Duggan C Wang CY Campbell KL Blackburn GL McTiernan A Dietary weight loss and exercise interventions effects on quality of life in overweight/obese postmenopausal women: a randomized controlled trial Int J Behav Nutr Phys Act 2011 8 118 10.1186/1479-5868-8-118 22026966 PMC3215656

[B41] Schulberg HC Coulehan JL Block MR Lave J Rodriguez E Scott CP Madonia MJ Imber S Perel J Clinical trials of primary care treatments for major depression: issues in design, recruitment and treatment Int J Psychiatry Med 1993 23 29 42 10.2190/TPTQ-M91J-6907-62RW 8514463

[B42] Cuijpers P Examining the effects of prevention programs on the incidence of new cases of mental disorders: the lack of statistical power Am J Psychiatry 2003 160 1385 1391 10.1176/appi.ajp.160.8.1385 12900296

[B43] Jha P Chaloupka FJ Corrao M Jacob B Reducing the burden of smoking world-wide: effectiveness of interventions and their coverage Drug Alcohol Rev 2006 25 597 609 10.1080/09595230600944511 17132576

[B44] Sharma S Zhuang Y Gomez-Pinilla F High-fat diet transition reduces brain DHA levels associated with altered brain plasticity and behaviour Sci Rep 2012 2 431 22666534 10.1038/srep00431 PMC3362800

[B45] Moylan S Maes M Wray NR Berk M The neuroprogressive nature of major depressive disorder: pathways to disease evolution and resistance, and therapeutic implications Mol Psychiatry 2012 in press 10.1038/mp.2012.33 22525486

[B46] Lopez-Garcia E Schulze MB Fung TT Meigs JB Rifai N Manson JE Hu FB Major dietary patterns are related to plasma concentrations of markers of inflammation and endothelial dysfunction Am J Clin Nutr 2004 80 1029 1035 15447916 10.1093/ajcn/80.4.1029

[B47] Duivis HE de Jonge P Penninx BW Na BY Cohen BE Whooley MA Depressive symptoms, health behaviors, and subsequent inflammation in patients with coronary heart disease: prospective findings from the heart and soul study Am J Psychiatry 2011 168 913 920 10.1176/appi.ajp.2011.10081163 21724664

[B48] Pasco JA Nicholson GC Williams LJ Jacka FN Henry MJ Kotowicz MA Schneider HG Leonard BE Berk M Association of high-sensitivity C-reactive protein with de novo major depression Br J Psychiatry 2010 197 372 377 10.1192/bjp.bp.109.076430 21037214

[B49] Soczynska JK Kennedy SH Woldeyohannes HO Liauw SS Alsuwaidan M Yim CY McIntyre RS Mood disorders and obesity: understanding inflammation as a pathophysiological nexus Neuromolecular Med 2011 13 93 116 10.1007/s12017-010-8140-8 21165712

[B50] Renn BN Feliciano L Segal DL The bidirectional relationship of depression and diabetes: a systematic review Clin Psychol Rev 2011 31 1239 1246 10.1016/j.cpr.2011.08.001 21963669

[B51] Maes M Depression is an inflammatory disease, but cell-mediated immune activation is the key component of depression Prog Neuropsychopharmacol Biol Psychiatry 2011 35 664 675 10.1016/j.pnpbp.2010.06.014 20599581

[B52] World Health Organization (WHO) Global status report on noncommunicable diseases 2010 2011 Geneva: WHO

[B53] Gortmaker SL Swinburn BA Levy D Carter R Mabry PL Finegood DT Huang T Marsh T Moodie ML Changing the future of obesity: science, policy, and action Lancet 2011 378 838 847 10.1016/S0140-6736(11)60815-5 21872752 PMC3417037

[B54] Merikangas KR He JP Burstein M Swanson SA Avenevoli S Cui L Benjet C Georgiades K Swendsen J Lifetime prevalence of mental disorders in U.S. adolescents: results from the National Comorbidity Survey Replication--Adolescent Supplement (NCS-A) J Am Acad Child Adolesc Psychiatry 2010 49 980 989 10.1016/j.jaac.2010.05.017 20855043 PMC2946114

[B55] Waters E de Silva-Sanigorski A Hall BJ Brown T Campbell KJ Gao Y Armstrong R Prosser L Summerbell CD Interventions for preventing obesity in children Cochrane Database Syst Rev 2011 12 CD001871 22161367 10.1002/14651858.CD001871.pub3

[B56] Vos T Carter R Barendregt J Mihalopoulos C Veerman L Magnus A Cobiac L Bertram M Wallace A Team AP Assessing Cost-Effectiveness in Prevention (ACE-Prevention): final report 2010 Melbourne: University of Queensland, Brisbane and Deakin University

[B57] Jacka F Berk M Depression, diet and exercise MJA Open 2012 Suppl 4 21 23 10.5694/mja12.10508 25370279

[B58] Collins PY Patel V Joestl SS March D Insel TR Daar AS Anderson W Dhansay MA Phillips A Shurin S Walport M Ewart W Savill SJ Bordin IA Costello EJ Durkin M Fairburn C Glass RI Hall W Huang Y Hyman SE Jamison K Kaaya S Kapur S Kleinman A Ogunniyi A Otero-Ojeda A Poo MM Ravindranath V Sahakian BJ Grand challenges in global mental health Nature 2011 475 27 30 10.1038/475027a 21734685 PMC3173804

